# Increased β-adrenergic stimulation augments vascular smooth muscle cell calcification via PKA/CREB signalling

**DOI:** 10.1007/s00424-021-02621-3

**Published:** 2021-09-26

**Authors:** Barbara Moser, Florian Poetsch, Misael Estepa, Trang T. D. Luong, Burkert Pieske, Florian Lang, Ioana Alesutan, Jakob Voelkl

**Affiliations:** 1 Institute for Physiology and Pathophysiology, Johannes Kepler University Linz, Altenberger Strasse 69, 4040 Linz, Austria; 2 Department of Internal Medicine and Cardiology, Charité – Universitätsmedizin Berlin, Campus Virchow-Klinikum, Berlin, Germany; 3 DZHK (German Centre for Cardiovascular Research), Partner Site Berlin, Berlin, Germany; 4 Department of Internal Medicine and Cardiology, German Heart Center Berlin (DHZB), Berlin, Germany; 5 Berlin Institute of Health (BIH), Berlin, Germany; 6 Department of Physiology I, Eberhard-Karls University Tübingen, Tübingen, Germany; 7 Department of Nephrology and Medical Intensive Care, Charité – Universitätsmedizin Berlin, Berlin, Germany

**Keywords:** β-adrenergic receptor, Isoproterenol, Protein kinase A, Phosphate, Vascular calcification, Vascular smooth muscle cells, Sympathetic overactivity, CREB

## Abstract

In chronic kidney disease (CKD), hyperphosphatemia promotes medial vascular calcification, a process augmented by osteogenic transdifferentiation of vascular smooth muscle cells (VSMCs). VSMC function is regulated by sympathetic innervation, and these cells express α- and β-adrenergic receptors. The present study explored the effects of β2-adrenergic stimulation by isoproterenol on VSMC calcification. Experiments were performed in primary human aortic VSMCs treated with isoproterenol during control or high phosphate conditions. As a result, isoproterenol dose dependently up-regulated the expression of osteogenic markers core-binding factor α-1 (*CBFA1*) and tissue-nonspecific alkaline phosphatase (*ALPL*) in VSMCs. Furthermore, prolonged isoproterenol exposure augmented phosphate-induced calcification of VSMCs. Isoproterenol increased the activation of PKA and CREB, while knockdown of the PKA catalytic subunit α (*PRKACA*) or of *CREB1* genes was able to suppress the pro-calcific effects of isoproterenol in VSMCs. β2-adrenergic receptor silencing or inhibition with the selective antagonist ICI 118,551 blocked isoproterenol-induced osteogenic signalling in VSMCs. The present observations imply a pro-calcific effect of β2-adrenergic overstimulation in VSMCs, which is mediated, at least partly, by PKA/CREB signalling. These observations may support a link between sympathetic overactivity in CKD and vascular calcification.

## Introduction

Chronic kidney disease (CKD) is associated with high mortality due to cardiovascular disease [23, 71]. The excessive cardiovascular risk in CKD patients may be fostered by medial vascular calcification [45, 71, 73], which promotes stiffening of vessels, subsequently leading to cardiac and circulatory dysfunctions [45, 71]. Vascular calcification is an active process involving transdifferentiation of vascular smooth muscle cells (VSMCs) into cells with some osteo-/chondroblast-like properties, characterized by expression of osteogenic transcription factors and enzymes [4, 15, 73]. Core-binding factor α-1 (CBFA1/RUNX2) is an osteogenic transcription factor essential for vascular calcification [60, 62], which induces expression of osteogenic enzymes, including tissue-nonspecific alkaline phosphatase (ALPL) [21, 62], a key effector of vascular mineralization [21, 73]. In CKD, hyperphosphatemia is considered the key pathological factor triggering osteogenic transdifferentiation of VSMCs and vascular calcification [73]. Nonetheless, additional pathological factors contribute to the regulation of vascular calcification in CKD [1–3, 28, 29, 56, 69, 75].

CKD has been considered a hyperadrenergic state [41] with sympathetic overactivity [13, 22, 42]. Sympathetic overactivity may be involved in the progression of renal disease and its cardiovascular complications [25, 34, 40, 55]. Chronic high sympathetic activity leads to overstimulation of adrenergic receptors promoting the development of cardiac and vascular diseases [52, 58, 66, 70]. Sympathetic blockade by stellate ganglion block reduces vascular calcification in a rat model [26]. Norepinephrine augments the calcification of VSMCs [26]. Besides α-adrenergic receptors associated with vasoconstriction, β-adrenergic receptors are expressed in VSMCs [18, 57, 63]. β2-adrenergic stimulation plays an important role in VSMC physiology and vascular dilation [12, 63]. β2-adrenergic signalling declines with increasing age, which has been discussed as a putative contributing factor for cardiovascular diseases in the elderly [57]. Furthermore, β2-adrenergic stimulation exhibits reno-protective effects [5]. However, the role of β-adrenergic overstimulation in the vasculature is less studied [68]. Chronic overstimulation of vascular β2-adrenergic receptors may induce oxidative stress and vascular dysfunction [16, 18].

Therefore, the present study explores a putative modulating role of β2-adrenergic activation by isoproterenol during phosphate-induced osteogenic transdifferentiation and calcification of VSMCs in vitro.

## Materials and methods

### Primary human aortic smooth muscle cells (HAoSMCs)

HAoSMCs (Fisher Scientific and Sigma-Aldrich) were routinely cultured as described previously [1, 28, 75] and used in experiments up to passage 12. *N* indicates the number of independent experiments performed at different passages of the cells. HAoSMCs were treated with the indicated concentrations or 1 μM isoproterenol (stock in PBS; Sigma-Aldrich) [70], 2 mM β-glycerophosphate (Sigma-Aldrich) [69, 74] or 1 μM ICI 118,551 (stock in DMSO; MedChemExpress) [78]. Equal amounts of vehicle were used as control. For calcification analysis, HAoSMCs were treated with calcification medium supplemented with 10 mM β-glycerophosphate and 1.5 mM CaCl_2_ (Sigma-Aldrich) [28, 75]. For long-term treatments, fresh medium with agents was added every 2–3 days. Where indicated, HAoSMCs were transfected with 10 nM PRKACA siRNA (ID:s11066), 10 nM CREB1 siRNA (ID:s3490), 10 nM ADRB2 siRNA (ID:s1122), or 10 nM negative control siRNA (ID:4390843) by using siPORT amine transfection agent (all from Fisher Scientific) [28].

### RNA isolation and RT-PCR

Total RNA was isolated from HAoSMCs by using Trizol reagent (Fisher Scientific). cDNA was synthesized with SuperScript III Reverse Transcriptase and oligo(dT)_12–18_ primers (Fisher Scientific). RT-PCR was performed in duplicate with iQ™ Sybr Green Supermix (Bio-Rad Laboratories) and CFX96 Real-Time PCR Detection System (Bio-Rad Laboratories). The specificity of the PCR products was confirmed by analysis of the melting curves. Relative mRNA expression was calculated by the 2^−ΔΔCt^ method using GAPDH as housekeeping gene, normalized to the control group. The following human primers were used (Fisher Scientific) [1, 28, 56, 76]:

- *ACTA2* fw: AAAAGACAGCTACGTGGGTGA
- *ACTA2* rev: GCCATGTTCTATCGGGTACTTC
- *ADRB1* fw: ATCGAGACCCTGTGTGTCATT
- *ADRB1* rev: GTAGAAGGAGACTACGGACGAG
- *ADRB2* fw: TGGTGTGGATTGTGTCAGGC
- *ADRB2* rev: GGCTTGGTTCGTGAAGAAGTC
- *ADRB3* fw: GACCAACGTGTTCGTGACTTC
- *ADRB3* rev: GCACAGGGTTTCGATGCTG
- *ALPL* fw: GGGACTGGTACTCAGACAACG
- *ALPL* rev: GTAGGCGATGTCCTTACAGCC
- *CBFA1* fw: GCCTTCCACTCTCAGTAAGAAGA
- *CBFA1* rev: GCCTGGGGTCTGAAAAAGGG
- *CD68* fw: CTTCTCTCATTCCCCTATGGACA
- *CD68* rev: GAAGGACACATTGTACTCCACC
- *CREB1* fw: CCACTGTAACGGTGCCAACT
- *CREB1* rev: GCTGCATTGGTCATGGTTAATGT
- *GAPDH* fw: GAGTCAACGGATTTGGTCGT
- *GAPDH* rev: GACAAGCTTCCCGTTCTCAG
- *LGALS3* fw: GTGAAGCCCAATGCAAACAGA
- *LGALS3* rev: AGCGTGGGTTAAAGTGGAAGG
- *PRKACA* fw: ACCCTGAATGAAAAGCGCATC
- *PRKACA* rev: CGTAGGTGTGAGAACATCTCCC
- *TAGLN* fw: CCGTGGAGATCCCAACTGG
- *TAGLN* rev: CCATCTGAAGGCCAATGACAT

### Protein isolation and Western blotting

HAoSMCs were lysed with ice-cold Pierce IP lysis buffer (Fisher Scientific; 25 mM Tris•HCl pH 7.4, 150 mM NaCl, 1% NP-40, 1 mM EDTA, 5% glycerol) supplemented with complete protease and phosphatase inhibitors cocktail (Fisher Scientific). Protein concentration was measured by the Bradford assay (Bio-Rad Laboratories). Equal amounts of proteins were boiled in Roti-Load1 Buffer (Carl Roth) at 100 °C for 10 min, separated on SDS–polyacrylamide gels and transferred to PVDF membranes. The membranes were incubated with primary rabbit: anti-ALPL (1:1000; Abcam, ab65834), anti-phospho-PKA substrate (RRXS*/T*)(1:1000; Cell Signalling, 9624), anti-phospho-CREB (Ser^133^)(1:1000; Cell Signalling, 9198), anti-CREB (1:1000; Cell Signalling, 9197), or anti-GAPDH (1:1000; Cell Signalling, 2118) antibodies at 4 °C overnight and then with secondary anti-rabbit HRP-conjugated antibody (1:1000, Cell Signalling) at room temperature for 1 h. The membranes were stripped in stripping buffer (Fisher Scientific) at room temperature. Bands were detected with Clarity Western ECL substrate (Bio-Rad Laboratories) and ChemiDoc MP imaging system (Bio-Rad Laboratories) and quantified by using the ImageJ software. For quantification of the phospho-PKA substrate signal, all bands per lane were used. Results are shown as the ratio of phosphorylated proteins/GAPDH, of phosphorylated/total protein, and of total protein/GAPDH, normalized to the control group [28, 74, 75].

### ALP activity assay

ALP activity in HAoSMCs was determined by using a colorimetric assay kit (Abcam). Results are shown normalized to total protein concentration measured by the Bradford assay (Bio-Rad Laboratories) [69] and to the control group.

### Calcium content analysis

HAoSMCs were decalcified in 0.6 M HCl at 4 °C overnight, and calcium content in the supernatant was determined by using the QuantiChrom Calcium assay kit (BioAssay Systems). HAoSMCs were lysed with 0.1 M NaOH/0.1% SDS, and protein concentration was measured by the Bradford assay (Bio-Rad Laboratories) [14, 44, 46, 64]. Results are shown normalized to total protein concentration [1, 28, 56] and to the control group.

### Calcium near-infrared (NIR) fluorescent imaging and Alizarin red staining

HAoSMCs were stained with OsteoSense 680 EX (1:100 dilution in medium, Perkin Elmer) overnight at 37 °C [49]. Images were collected using the ChemiDoc MP imaging system (Bio-Rad Laboratories) with excitation/emission (bandpass) wavelengths of 680/715(30) nm for detecting the calcium NIR signal (red) and with excitation/emission (bandpass) wavelengths of 488/532(28) nm as control (green, plate autofluorescence). HAoSMCs were fixed with 4% paraformaldehyde/PBS and stained with 2% Alizarin red (pH 4.5). The calcified areas are shown as red staining.

### Statistics

Data are shown as scatter dot plots and arithmetic means ± SEM. Normality was tested with Shapiro–Wilk test. Non-normal datasets were transformed (log, reciprocal, or sqrt) prior to statistical testing to provide normality. Statistical testing was performed by one-way ANOVA followed by Tukey (homoscedastic data) or Games-Howell (heteroscedastic data) post hoc test and by the Steel–Dwass method (non-normal data). Two groups were compared by unpaired two-tailed *t*-test or Mann–Whitney *U*-test. *p* < 0.05 was considered statistically significant.

## Results

**Fig. 1:**
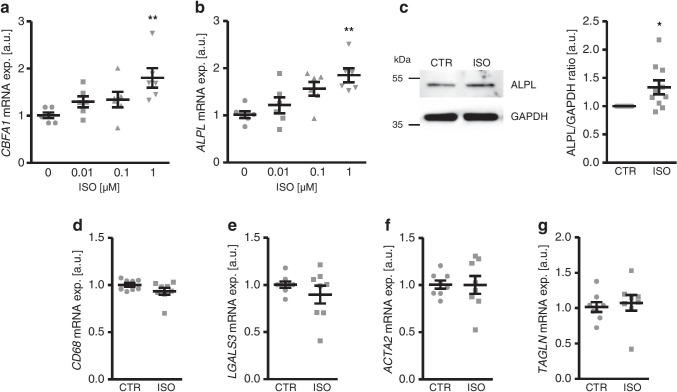
Isoproterenol up-regulates the expression of osteogenic markers in HAoSMCs. **a**, **b** Scatter dot plots and arithmetic means ± SEM (*n* = 6; arbitrary units, a.u.) of *CBFA1* (**a**) and *ALPL* (**b**) relative mRNA expression in HAoSMCs following treatment for 24 h with the indicated concentrations of isoproterenol (ISO, 0–1 μM). **c** Representative Western blots and scatter dot plots and arithmetic means ± SEM (*n* = 10; a.u.) of normalized ALPL/GAPDH protein ratio in HAoSMCs following treatment for 7 days with 1 μM isoproterenol (ISO). **d–g** Scatter dot plots and arithmetic means ± SEM (*n* = 8; a.u.) of *CD68* (**d**), *LGALS3* (**e**), *ACTA2* (**f**), and *TAGLN* (**g**) relative mRNA expression in HAoSMCs following treatment for 24 h with 1 μM isoproterenol (ISO). *(*p* < 0.05), **(*p* < 0.01) statistically significant vs control HAoSMCs

A first series of experiments investigated the effects of chronic β-adrenergic stimulation on markers of osteogenic transdifferentiation of VSMCs. To this end, HAoSMCs were treated with increasing concentrations of isoproterenol, a nonspecific β-adrenergic receptor agonist, and the expression of osteogenic transcription factor *CBFA1* and osteogenic enzyme *ALPL* was determined. As shown in Fig. 1a and b, isoproterenol treatment up-regulated *CBFA1* and *ALPL* mRNA expression in HAoSMCs in a concentration-dependent manner, effects reaching statistical significance at 1 μM isoproterenol concentration. Accordingly, prolonged isoproterenol treatment significantly increased ALPL protein abundance in HAoSMCs (Fig. 1c). In contrast, exposure of HAoSMCs to isoproterenol did not significantly modify macrophage markers *CD68* and *LGALS3* or smooth muscle markers *ACTA2* and *TAGLN* mRNA expression (Fig. 1d–g).

**Fig. 2:**
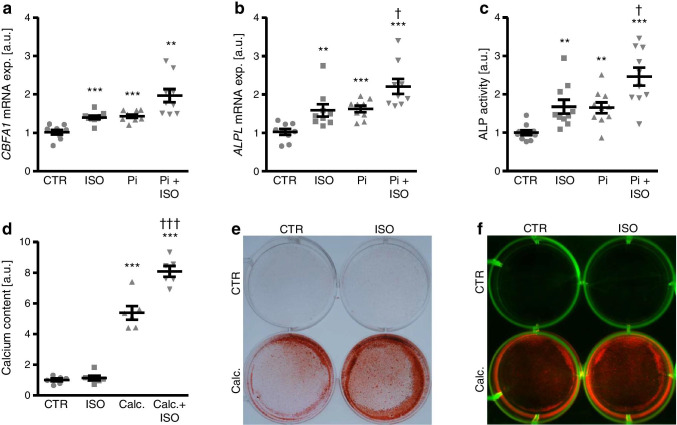
Isoproterenol aggravates calcification of HAoSMCs during high phosphate conditions. **a, b** Scatter dot plots and arithmetic means ± SEM (*n* = 9; arbitrary units, a.u.) of *CBFA1* (**a**) and *ALPL* (**b**) relative mRNA expression in HAoSMCs following treatment for 24 h with control (CTR) or 1 μM isoproterenol (ISO) without and with 2 mM β-glycerophosphate (Pi). **c** Scatter dot plots and arithmetic means ± SEM (*n* = 10; a.u.) of normalized ALP activity in HAoSMCs following treatment for 7 days with control (CTR) or 1 μM isoproterenol (ISO) without and with 2 mM β-glycerophosphate (Pi). **d** Scatter dot plots and arithmetic means ± SEM (*n* = 6; a.u.) of normalized calcium content in HAoSMCs following treatment for 11 days with control (CTR) or 1 μM isoproterenol (ISO) without and with calcification medium (Calc.; 10 mM β-glycerophosphate + 1.5 mM CaCl_2_). **(*p* < 0.01), ***(*p* < 0.001) statistically significant vs control HAoSMCs; †(*p* < 0.05), †††(*p* < 0.001) statistically significant vs Pi-/Calc.-treated HAoSMCs. **e** Representative images showing Alizarin red staining in HAoSMCs following treatment for 11 days with control (CTR) or 1 μM isoproterenol (ISO) without and with calcification medium (Calc.; 10 mM β-glycerophosphate + 1.5 mM CaCl_2_). Calcified areas: red staining. **f** Representative images showing calcium NIR fluorescent staining in HAoSMCs following treatment for 11 days with control (CTR) or 1 μM isoproterenol (ISO) without and with calcification medium (Calc.; 10 mM β-glycerophosphate + 1.5 mM CaCl_2_). Calcified areas: red; plate autofluorescence: green

Next experiments explored the effects of isoproterenol on osteogenic markers *CBFA1* and *ALPL* expression as well as calcification of HAoSMCs during elevated phosphate conditions. Isoproterenol treatment up-regulated osteogenic markers mRNA expression in HAoSMCs to similarly high levels as following exposure to the phosphate donor β-glycerophosphate (Fig. 2a, b). Additional treatment with isoproterenol tended to enhance β-glycerophosphate-induced *CBFA1* mRNA expression, a difference, however, not reaching statistical significance (*p* = 0.057; Fig. 2a), and significantly augmented β-glycerophosphate-induced *ALPL* mRNA expression in HAoSMCs (Fig. 2b). Similarly, isoproterenol significantly increased ALP activity in HAoSMCs during control as well as elevated phosphate conditions (Fig. 2c). As shown by quantification of calcium content, isoproterenol did not significantly modify calcification of HAoSMCs during control conditions, but significantly aggravated mineralization induced by a calcification medium (Fig. 2d). These effects were confirmed by Alizarin red staining (Fig. 2e) and calcium NIR fluorescent imaging (Fig. 2f) showing an enhancement of calcification induced by calcification medium in the presence of isoproterenol. Taken together, β-adrenergic stimulation with isoproterenol aggravated phosphate-induced HAoSMC osteogenic marker expression and calcification in vitro.

**Fig. 3:**
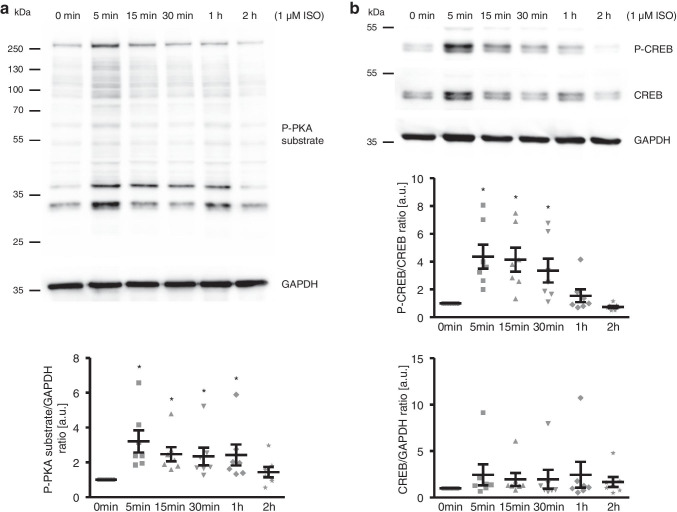
Isoproterenol increases PKA and CREB activation in HAoSMCs. Representative Western blots and scatter dot plots and arithmetic means ± SEM (*n* = 7; arbitrary units, a.u.) of normalized phospho-PKA substrate/GAPDH protein ratio (**a**) and phospho-CREB/CREB and CREB/GAPDH protein ratios (**b**) in HAoSMCs following treatment for the indicated times (0–2 h) with 1 μM isoproterenol (ISO). *(*p* < 0.05) statistically significant vs control HAoSMCs

To elucidate the underlying mechanisms of the pro-calcific effects of isoproterenol, the possible involvement of protein kinase A (PKA)/cAMP-responsive element-binding protein (CREB) signalling was investigated. As shown by Western blotting, isoproterenol significantly up-regulated the phosphorylation at RRXS*/T* substrate motif for PKA (where R refers to arginine, X refers to any amino acid, and S and T represent serine and threonine residue, respectively) of total cellular proteins and, thus, PKA activity in HAoSMCs following 5 min of treatment, levels remaining significantly higher up to 1 h of treatment (Fig. 3a). Similarly, isoproterenol increased the activation of CREB in HAoSMCs, as shown by increased phosphorylation at Ser^133^, following 5 min and up to 30 min of treatment without significantly affecting total CREB protein abundance (Fig. 3b).

**Fig. 4:**
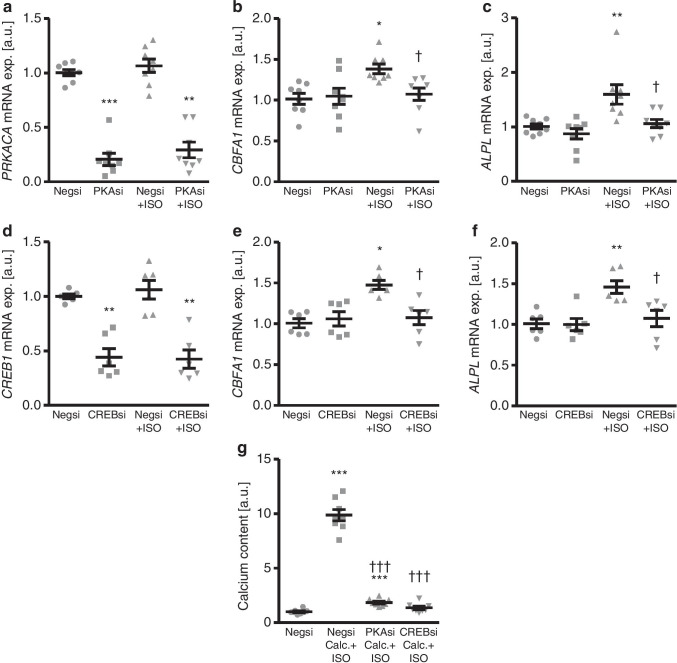
Silencing of PKA or CREB suppresses the pro-calcific effects of isoproterenol in HAoSMCs. **a** Scatter dot plots and arithmetic means ± SEM (*n* = 8; arbitrary units, a.u.) of *PRKACA* relative mRNA expression in HAoSMCs following transfection with negative control (Negsi) or PRKACA (PKAsi) siRNA and treatment for 24 h with control or 1 μM isoproterenol (ISO). **(*p* < 0.01), ***(*p* < 0.001) statistically significant vs Negsi-transfected HAoSMCs. **b**, **c** Scatter dot plots and arithmetic means ± SEM (*n* = 8; a.u.) of *CBFA1* (**b**) and *ALPL* (**c**) relative mRNA expression in HAoSMCs following transfection with negative control (Negsi) or PRKACA (PKAsi) siRNA and treatment for 24 h with control or 1 μM isoproterenol (ISO). *(*p* < 0.05), **(*p* < 0.01) statistically significant vs Negsi-transfected HAoSMCs; †(*p* < 0.05) statistically significant vs Negsi-transfected ISO-treated HAoSMCs. **d** Scatter dot plots and arithmetic means ± SEM (*n* = 6; a.u.) of *CREB1* relative mRNA expression in HAoSMCs following transfection with negative control (Negsi) or CREB1 (CREBsi) siRNA and treatment for 24 h with control or 1 μM isoproterenol (ISO). **(*p* < 0.01) statistically significant vs Negsi-transfected HAoSMCs. **e**, **f** Scatter dot plots and arithmetic means ± SEM (*n* = 6; a.u.) of *CBFA1* (**e**) and *ALPL* (**f**) relative mRNA expression in HAoSMCs following transfection with negative control (Negsi) or CREB1 (CREBsi) siRNA and treatment for 24 h with control or 1 μM isoproterenol (ISO). *(*p* < 0.05), **(*p* < 0.01) statistically significant vs Negsi-transfected HAoSMCs; †(*p* < 0.05) statistically significant vs Negsi-transfected ISO-treated HAoSMCs. **g** Scatter dot plots and arithmetic means ± SEM (*n* = 8; a.u.) of normalized calcium content in HAoSMCs following transfection with negative control (Negsi), PRKACA (PKAsi), or CREB1 (CREBsi) siRNA and treatment for 11 days with control (CTR) or 1 μM isoproterenol (ISO) and calcification medium (Calc.; 10 mM β-glycerophosphate + 1.5 mM CaCl_2_). ***(*p* < 0.001) statistically significant vs Negsi-transfected HAoSMCs; †††(*p* < 0.001) statistically significant vs Negsi-transfected Calc. + ISO-treated HAoSMCs

Furthermore, suppressing the endogenous expression in HAoSMCs of the PKA catalytic subunit α by silencing of the *PRKACA* gene using small interfering RNA (siRNA) (Fig. 4a) was able to significantly blunt isoproterenol-induced *CBFA1* and *ALPL* mRNA expression (Fig. 4b, c). In addition, knockdown of the *CREB1* gene by siRNA (Fig. 4d) significantly suppressed isoproterenol-induced osteogenic markers mRNA expression in HAoSMCs (Fig. 4e, f). *PRKACA* (Fig. 4a), or *CREB1* (Fig. 4d) mRNA expression was not significantly affected by isoproterenol treatment. More importantly, silencing of the *PRKACA* or *CREB1* genes significantly reduced calcification of HAoSMCs induced by isoproterenol together with calcification medium (Fig. 4g). Thus, PKA and CREB are activated by isoproterenol and participate in isoproterenol-induced osteogenic signalling and augmentation of calcification in HAoSMCs.

**Fig. 5:**
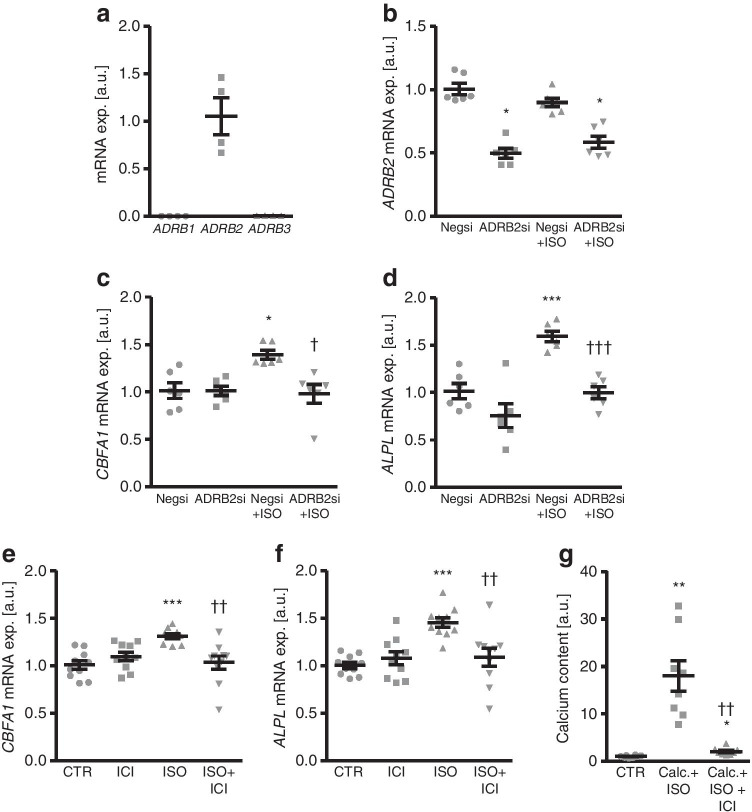
Knockdown or blockade of the β2-adrenergic receptor blunts the pro-calcific effects of isoproterenol in HAoSMCs. **a** Scatter dot plots and arithmetic means ± SEM (*n* = 4; arbitrary units, a.u.) of β-adrenergic receptors *ADRB1*, *ADRB2*, and *ADRB3* relative mRNA expression in HAoSMCs. **b** Scatter dot plots and arithmetic means ± SEM (*n* = 6; a.u.) of *ADRB2* relative mRNA expression in HAoSMCs following transfection with negative control (Negsi) or ADRB2 (ADRB2si) siRNA and treatment for 24 h with control or 1 μM isoproterenol (ISO). *(*p* < 0.05) statistically significant vs Negsi-transfected HAoSMCs. **c**, **d** Scatter dot plots and arithmetic means ± SEM (*n* = 6; a.u.) of *CBFA1* (**c**) and *ALPL* (**d**) relative mRNA expression in HAoSMCs following transfection with negative control (Negsi) or ADRB2 (ADRB2si) siRNA and treatment for 24 h with control or 1 μM isoproterenol (ISO). *(*p* < 0.05), ***(*p* < 0.001) statistically significant vs Negsi-transfected HAoSMCs; †(*p* < 0.05), †††(*p* < 0.001) statistically significant vs Negsi-transfected ISO-treated HAoSMCs. **e**, **f** Scatter dot plots and arithmetic means ± SEM (n = 10; a.u.) of *CBFA1* (**e**) and *ALPL* (**f**) relative mRNA expression in HAoSMCs following treatment for 24 h with control (CTR) or 1 μM isoproterenol (ISO) without and with 1 μM β2-adrenergic receptor antagonist ICI 118,551 (ICI). **g** Scatter dot plots and arithmetic means ± SEM (*n* = 8; a.u.) of normalized calcium content in HAoSMCs following treatment for 11 days with control (CTR) or 1 μM isoproterenol (ISO) and calcification medium (Calc.; 10 mM β-glycerophosphate + 1.5 mM CaCl_2_) without and with 1 μM β2-adrenergic receptor antagonist ICI 118,551 (ICI). *(*p* < 0.05), **(*p* < 0.01), ***(*p* < 0.001) statistically significant vs control HAoSMCs; ††(*p* < 0.01), statistically significant vs ISO-/Calc. + ISO-treated HAoSMCs

To identify the β-adrenergic receptor mediating the osteoinductive effects of isoproterenol in HAoSMCs, the mRNA levels of the β-adrenergic receptor subtypes were determined. In accordance with the previous reports [57], the β2-adrenergic receptor, encoded by the *ADRB2* gene, had the highest relative expression in HAoSMCs (Fig. 5a). Next, the endogenous expression of the β2-adrenergic receptor in HAoSMCs was suppressed by silencing of the *ADRB2* gene using siRNA followed by additional treatment without or with isoproterenol. As a result, *ADRB2* mRNA expression was significantly reduced in ADRB2 siRNA-transfected HAoSMCs as compared to negative control siRNA-transfected HAoSMCs, levels not significantly affected by additional isoproterenol treatment (Fig. 5b). Moreover, isoproterenol significantly up-regulated osteogenic markers mRNA expression in control-transfected HAoSMCs, effects significantly suppressed by *ADRB2* silencing (Fig. 5c, d). Furthermore, additional treatment with the highly selective β2-adrenergic receptor antagonist ICI 118,551 significantly blunted isoproterenol-induced osteogenic markers mRNA expression (Fig. 5e, f) and significantly reduced calcification of HAoSMCs induced by isoproterenol together with calcification medium (Fig. 5g). Thus, the β2-adrenergic receptor mediated, at least partly, the pro-calcific effects of isoproterenol in HAoSMCs.

## Discussion

The present study identifies a promoting effect of chronic β-adrenergic stimulation by isoproterenol through the β2-adrenergic receptor on osteogenic marker expression and calcification of VSMCs. These observations seem surprising, since β2-adrenergic activation ameliorates calcification in valvular cells [53]. Furthermore, acute β2-adrenergic receptor activation induces vasodilation [11], an effect that may be impaired by aging or atherosclerosis [36, 57]. In contrast, chronic isoproterenol exposure augments vasoconstrictor responses and induces vascular dysfunction [18, 19, 68]. Injections of high doses of adrenaline induce microcrystalline calcifications in aortic mitochondria of rabbits [7]. Also, repeated isoproterenol injections in rabbits induce oxidative stress and DNA damage in cerebral artery VSMCs [39]. Although further studies on the vascular effects of β-adrenergic activation are required, β-adrenergic overstimulation has been discussed as a putative factor in sympathetic overactivity [68].

Isoproterenol treatment of VSMCs promotes the expression of osteogenic markers *CBFA1* and *ALPL*. These effects appear to be mediated through the β2-adrenergic receptor and are blocked by a selective antagonist. The pro-calcifying effects of isoproterenol can also be abrogated by silencing of the β2-adrenergic receptor, which did not abolish, but only reduce *ADRB2* expression. Thus, it is tempting to speculate that not physiological activation but unphysiological overactivation of the downstream signalling pathways may be responsible for the pro-calcific effects of the β2-adrenergic receptor. These downstream effects of β2-adrenergic stimulation on osteogenic marker expression involve PKA and CREB. Although β2-adrenergic signalling events in VSMC are complex, sustained PKA activation has been identified after isoproterenol exposure [31]. Activation of PKA by TNF-α induces VSMC calcification, suggesting a detrimental role of chronic PKA activation in vascular calcification [32, 33, 65]. PKA has also been implied in the osteogenic effects of uremic serum on VSMC calcification [9]. Accordingly, PKA inhibition attenuates osteogenic transdifferentiation and calcification of VSMCs [37, 65] or aortic rings [67]. However, the role of PKA may be more complex, since also anti-calcific effects of PKA due to inhibition of endoplasmic reticulum stress are described [8].

A downstream target of PKA, activated by Ser^133^ phosphorylation, is the transcription factor CREB [48]. CREB has been shown to activate CBFA1/RUNX2 [79], and activation of the PKA/CREB pathway plays an important role in osteogenic differentiation of bone marrow stromal cells [59] and mesenchymal stem cells [80]. CREB has also been implied as a factor downstream of PKA-promoting vascular calcification [6, 65]. Furthermore, CREB mediates the pro-calcific effects of low potassium conditions in VSMCs [61]. CREB may also be involved in the pro-calcific effects of transforming growth factor β1 [27]. The present observations show a transient activation of PKA/CREB after isoproterenol exposure. The subsequent downstream effects of this CREB activation to induce a pro-calcific phenotype after isoproterenol exposure are currently unclear. CREB may modulate autophagy, which inherits a complex and important role in VSMC calcification [61]. CREB is further involved in pro-inflammatory signalling pathways in VSMCs [43]. After isoproterenol exposure, no alterations of the macrophage markers *LGALS3* and *CD68* or smooth muscle markers *ACTA2* and *TAGLN* are detectable. Nonetheless, the PKA/CREB pathway seems to be required for the full pro-calcific effects of isoproterenol, since silencing of CREB or PKA abrogates these effects. These observations cannot rule out other putative mechanisms of isoproterenol exposure on VSMC calcification. β-adrenergic stimulation may induce intracellular signalling leading to RANKL release [35], ERK1/2 activation [58], PI3K pathway activation [10], oxidative stress [54], inflammation [18, 30, 58], or apoptosis [30, 54], all known as regulators of VSMC osteogenic transdifferentiation and vascular calcification [51, 72, 73].

The effects of β2-adrenergic stimulation on VSMC calcification may be a relevant aspect in conditions of sympathetic hyperactivity, such as CKD [25, 40, 55], but more complex mechanisms may play a significant role. The current study is clearly limited by artificial VSMC culture conditions with isoproterenol administration, a condition different from the adrenergic system in the vasculature in vivo [47]. Isoproterenol treatment in cell culture would not mimic a rhythmicity of the sympathetic nervous system and its co-transmitters [47]. β2-adrenergic receptors are involved in the circadian oscillations of vascular adhesion molecules after TNF-α stimulation [20]. In endothelial cells, isoproterenol promotes nitric oxide (NO) release, an important inhibitor of vascular calcification [24, 73]. Mice deficient for the β2-adrenergic receptor exhibit reduced aortic NO production [17]. Overexpression of the β2-adrenergic receptor in endothelial progenitor cells improves vascular repair after vascular injury [38]. However, overstimulation with isoproterenol induces endothelial synthase uncoupling [68] and up-regulates expression of inflammatory cytokines in endothelial cells [50]. A complex immunomodulatory role of the β2-adrenergic receptor has been discussed [77]. Clearly, caution is warranted when interpreting the current results, especially for mechanisms of vascular calcification in human patients.

In conclusion, β-adrenergic overstimulation by isoproterenol aggravates phosphate-induced VSMC calcification in vitro, effects mediated, at least in part, by the β2-adrenergic receptor and involving PKA/CREB signalling activation. Further studies are required to determine a possible association of adrenergic stimulation and vascular calcification.
